# A Comparison of the Effectiveness of the McCoy Laryngoscope and the C-MAC D-Blade Video Laryngoscope in Obese Patients

**DOI:** 10.3390/medicina60081285

**Published:** 2024-08-09

**Authors:** Jung-Min Lee, Soo-Kyung Lee, Minsoo Jang, Minho Oh, Eun-Young Park

**Affiliations:** 1 Department of Anesthesiology and Pain Medicine, Cha Ilsan Medical Center, Cha University, 1205, Jungang-ro, Ilsandong-gu, Goyang-si 10414, Gyeonggi-do, Republic of Korea; 2 Department of Anesthesiology and Pain Medicine, Hallym University Sacred Heart Hospital, 22, Gwanpyeong-ro 170 beon-gil, Dongan-gu, Anyang-si 14068, Gyeonggi-do, Republic of Korea

**Keywords:** obesity, laryngoscopy, general anesthesia, airway management, intratracheal intubation

## Abstract

*Background and Objective:* Obesity is associated with difficult or failed intubation attempts, making general anesthesia challenging for anesthesiologists to perform. The purpose of this study was to evaluate and compare the efficacy of a McCoy laryngoscope and a C-MAC D-blade video laryngoscope for intubation in obese patients with a body mass index (BMI) ≥ 35 kg/m^2^. *Methods:* In total, 104 patients were randomly assigned to be intubated with a McCoy (McCoy group) or C-MAC D-blade video laryngoscope (C-MAC group). The primary outcome was intubation time. The secondary outcomes were vocal cord exposure time, vocal cord passage time, proportion of successful intubation, mask ventilation scale, intubation difficulty scale (IDS), percentage of glottis opening (POGO) score, and hemodynamic variables. *Results:* Although the intubation time did not significantly differ, the C-MAC group showed shorter vocal cord exposure times and a higher rate of successful vocal cord exposure within 5 s. The IDS value was significantly lower in the C-MAC group than in the McCoy group. The proportion of patients who required an increase in lifting force during laryngoscopy was higher in the McCoy group than in the C-MAC group, which may explain the difference in MAP between the groups. *Conclusions:* Both the McCoy laryngoscope and the C-MAC D-blade video laryngoscope were useful during the intubation of obese patients. The C-MAC D-blade video laryngoscope might be more useful for obese patients in terms of hemodynamic stability.

## 1. Introduction

General anesthesia requiring the intubation of obese patients is challenging for anesthesiologists, as obesity is associated with difficult or failed intubation attempts [1]. After injecting sedatives and muscle relaxants, anesthesiologists find it challenging to ventilate obese patients using a mask due to the excess soft tissue surrounding the chin and neck [2,3]. Furthermore, the functional reserve capacity is reduced in obese patients [1], and they are susceptible to rapid hypoxia during apnea events induced by a sedative, in the context of inadequate mask ventilation [4].

About 40% of patients with problematic airway management during the perioperative period, from induction to recovery, have obesity [5]. A high BMI (over 35 kg/m^2^) was found to be a significant predictor of difficult intubation in a large cohort study, despite some disagreement about the link between obesity and the incidence of difficult intubations [6]. A particularly broad circumference around the neck has been linked to challenging intubation [7,8]. By limiting the anterior movement of the pharyngeal structure, excessive fat tissue in the retropharynx or submandibular region may be the cause of an insufficient laryngoscopic view [5].

For those patients, the general guidelines include anticipating difficult ventilation/intubation through a thorough physical examination prior to induction, a preparing device (invasive airway or a laryngeal mask) that helps to maintain the patient’s oxygenation, and using an awake intubation technique based on the anesthesiologist’s judgement [5]. In the event of unexpected difficult intubation, the patient’s oxygenation should be maintained by initiating the difficult airway algorithm.

Various intubation devices other than the conventional Macintosh laryngoscope have been used to achieve the successful intubation of obese patients. The McCoy laryngoscope has a hinged tip that can be controlled by a lever attached to a handle. Intubation using a McCoy laryngoscope reportedly decreases the Cormack–Lehane grade and reduces the hemodynamic response to laryngoscopy, compared with intubation using the conventional Macintosh laryngoscope [9,10,11]. The C-MAC video laryngoscope has a camera/monitor system, in which the light source is attached to the blade tip, thereby providing a wide angle of view and a high-resolution color image [12]. Compared with the C-MAC Macintosh blade, the C-MAC D-blade is half-moon-shaped; the increased angulation of the blade facilitates successful outcomes when intubation is difficult [13]. The aim of the present study was to compare the clinical effectiveness of the McCoy laryngoscope and C-MAC D-blade video laryngoscope for intubation in obese patients.

## 2. Materials and Methods

This randomized trial was approved by the Institutional Review Board of Hallym University Sacred Heart Hospital (approval number 2019-10-010-001) and registered at ClinicalTrials.gov (NCT05146531). In total, 104 obese patients scheduled for general anesthesia requiring orotracheal intubation were enrolled in this study. The inclusion criteria were American Association of Anesthesiologists status II–III, body mass index (BMI) ≥ 35 kg/m^2^, and age 20–70 years. Patients with cervical spine injury, dental injury, pregnancy, or a history of tracheal diseases (e.g., trauma, tumor, and/or infection) were excluded. Written informed consent was obtained from all study participants. The patients underwent a brief assessment of medical history, followed by a physical examination by other anesthesiologists who did not perform intubation, which included mouth opening (distance between the upper and lower incisors), Mallampati classification (I—visible tonsil; tonsillar pillars and uvula; II—visible uvula and soft palate; III—visible soft palate only; IV—visible hard palate only), head and neck movement, ability for prognathism (yes—lower teeth can be moved in front of upper teeth; partial—lower teeth can be placed in line with upper teeth; no—lower teeth cannot be placed in line with upper teeth), thyromental distance (defined as the distance from the mentum to the thyroid notch with the head fully extended), neck circumstance (measured at the level of cricoid cartilage), and history of difficult intubation (via chart review or history taking) [14]. Immediately before the induction of anesthesia, the patients were randomly assigned to one of two groups—intubation with the McCoy laryngoscope (McCoy group, *n* = 52) or intubation with the C-MAC D-blade video laryngoscope (C-MAC group, *n* = 52)—using randomization site (http://www.random.org, accessed on 30 November 2019).

All participating patients fasted for 8 h before surgery. Patients were attached to standard monitoring equipment, including an electrocardiography device and monitors for noninvasive blood pressure and pulse oximetry. Hemodynamic variables were recorded before induction, immediately before intubation, immediately after intubation, and 3 min after intubation. After 5 min of preoxygenation with 100% oxygen, anesthesia was induced with intravenous propofol (2 mg/kg ideal body weight), via the continuous infusion of remifentanil (0.1–0.2 μg/kg/min lean body weight) and intravenous rocuronium (0.6 mg/kg ideal body weight). We also used a BIS monitor on all patients. All patients’ BIS scores were 40–60 two minutes after the rocuronium injection, and senior residents who had performed > 100 successful intubations using a McCoy laryngoscope and C-MAC D-blade video laryngoscope conducted intubation with one of two kinds of laryngoscope. The senior residents who conducted intubation also recorded the mask ventilation scale (Table 1) and intubation difficulty scale (IDS, Table 2) levels, as well as the percentage of glottis opening (POGO) score.

The primary outcome was intubation time, measured from the point at which the laryngoscope blade crossed the incisors until the first strike of the capnograph, as determined using the monitor. Secondary outcomes were vocal cord exposure time (measured from the point at which the laryngoscope blade crossed the incisors until the assistant passed the endotracheal tube to the anesthesiologist), vocal cord passage time (measured from the point at which the laryngoscope blade crossed the incisors until the assistant removed the stylet from the endotracheal tube), rate of successful intubation, hemodynamic variables, mask ventilation scale, IDS, and POGO score.

Patients who exhibited desaturation (SpO_2_ < 90%) or could not be intubated (>3 intubation attempts or intubation time > 90 s) were excluded from this study, and a difficult airway algorithm was implemented for resolution.

The sample size was estimated using G*Power software version 3.1.9.2, based on a pilot study of 20 obese patients conducted in our hospital; 82 patients were required to detect a 5 s difference in intubation time (effect size = 0.56; power = 80%; α = 0.05). Considering a potential dropout rate of 20%, 104 patients were included in this study.

Parametric data were analyzed using Student’s *t*-test, repeated measures analysis of variance, and the Pearson chi-squared test; nonparametric data were analyzed using the Mann–Whitney test, generalized estimating equation, and Fisher’s exact test. The rates of successful intubation were analyzed using the Kaplan–Meier method and log rank test. Statistical data were evaluated using IBM SPSS Statistics version 26.0 (IBM Corp., Armonk, NY, USA). The threshold of statistical significance was regarded as *p* < 0.05.

## 3. Results

**Figure 1:**
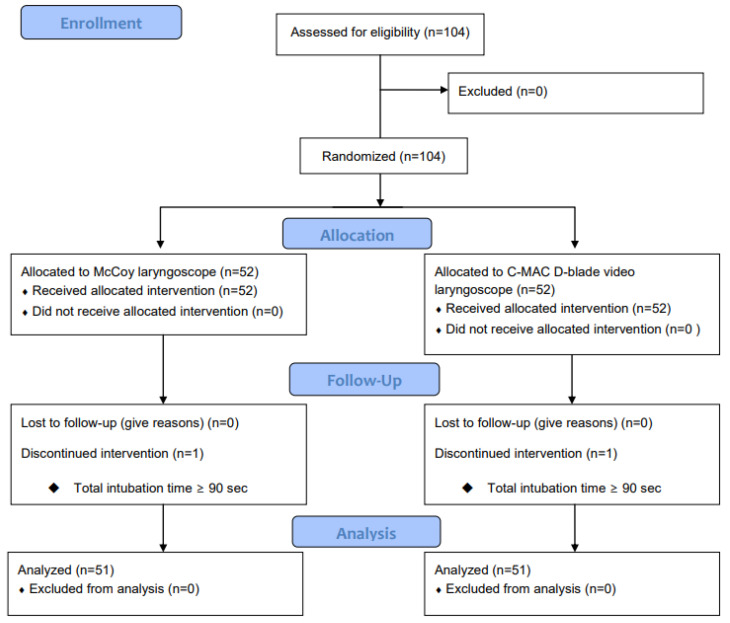
CONSORT flow chart.

Among the 104 patients enrolled, 1 patient from each group was excluded from the study because they could not be intubated within 90 s (Figure 1). There were no significant differences between the McCoy and C-MAC groups with respect to demographic data or airway assessment findings (Table 3).

**Figure 2:**
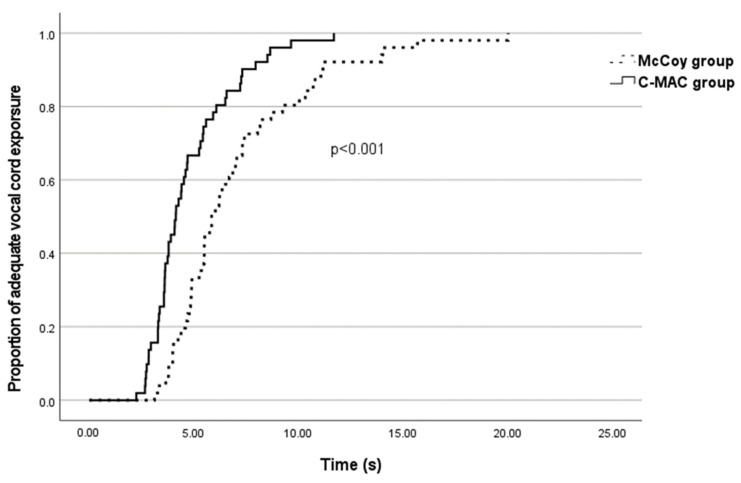
Cumulative proportions of adequate vocal cord exposure with McCoy laryngoscope and C-MAC D-blade video laryngoscope.

The intubation data are shown in Table 4. The mask ventilation scales were comparable between the two groups (*p* = 0.070). Although there was a significant difference between groups in vocal cord exposure time [McCoy, 5.85 (4.81–8.10) s vs. C-MAC, 4.14 (3.47–5.52) s; *p* < 0.001], the vocal cord passage time [McCoy, 10.76 (8.75–14.39) s vs. C-MAC, 10.33 (9.21–14.35) s; *p* = 0.756] and intubation time [McCoy, 32.54 (29.70–37.21) s vs. C-MAC, 30.72 (28.12–32.19) s; *p* = 0.499] did not significantly differ. The rates of successful vocal cord exposure within 5 s were 33.3% in the McCoy group (*n* = 17) and 66.7% in the C-MAC group (*n* = 34), indicating a significant difference according to Kaplan–Meier analysis and the log rank test (*p* < 0.001, Figure 2). The rates of successful intubation in 30 s were 29.4% (*n* = 15) in the McCoy group and 43.1% in the C-MAC group (*n* = 22).

The POGO scores in the McCoy and C-MAC groups were 70% (50–80%) and 70% (50–90%), respectively (*p* = 0.529). During intubation, no patients experienced tooth damage; however, two patients in the C-MAC group experienced mild lip trauma.

The IDS value significantly differed between the two groups [McCoy, 2 (1–3) vs. C-MAC, 1 (0–2); *p* = 0.046]; the frequency of increased lifting force was higher in the McCoy group than in the C-MAC group (*p* = 0.027, Table 5). In the McCoy group, two patients underwent two intubation attempts; the second anesthesiologist successfully conducted intubation after the first anesthesiologist had been unsuccessful in one patient. There were no differences between groups in the number of alternative techniques, Cormack–Lehane grade, application of laryngeal pressure, or vocal cord mobility (Table 5).

**Figure 3:**
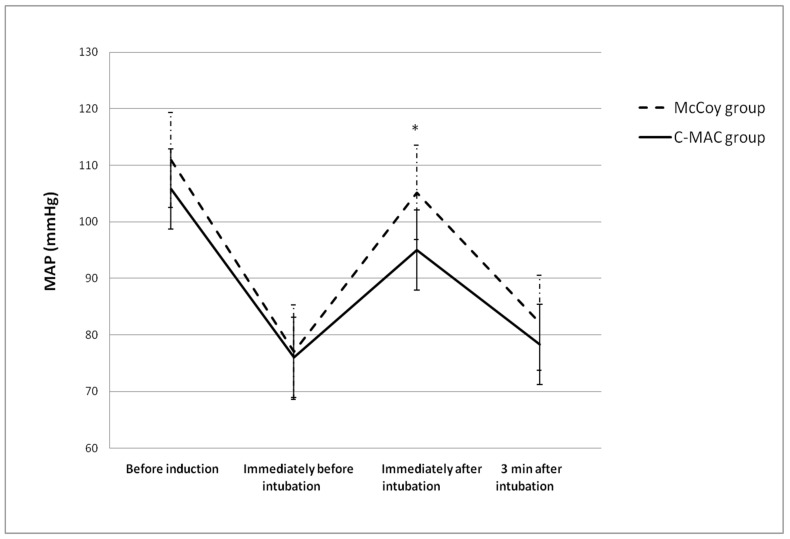
Changes in MAP over time in each group. Data are shown as mean ± standard deviation. MAP, mean arterial pressure. * *p* = 0.011 for McCoy vs. C-MAC at the same time point.

We also measured hemodynamic variables including mean arterial pressure (MAP), heart rate (HR), and saturation (SpO_2_) before induction, immediately before intubation, immediately after intubation, and 3 min after intubation (Table 6). There were no differences in HR (*p* = 0.974) or SpO_2_ (*p* = 0.775); however, MAP significantly differed over time (*p* = 0.042), particularly immediately after intubation (McCoy, 105.20 ± 22.40 mmHg vs. C-MAC, 95.02 ± 16.79 mmHg; *p* = 0.011, Figure 3).

## 4. Discussion

This study compared the clinical effectiveness of the McCoy laryngoscope and C-MAC D-blade video laryngoscope in obese patients with a BMI ≥ 35 kg/m^2^. There were no significant differences in vocal cord passage time, intubation time, or POGO score between the two groups; however, the C-MAC D-blade video laryngoscope showed superiority in terms of vocal cord exposure time and IDS indices. Among the IDS indices, the proportion of increased lifting force during laryngoscopy was higher in the McCoy group, and the MAP immediately after intubation was significantly higher in the McCoy group than in the C-MAC group.

For patients with anticipated difficult intubation, awake fiberoptic intubation is the gold-standard technique [5], but a learning curve period is needed for the technique. Supraglottic devices, such as the laryngeal mask airway (LMA) or the intubating laryngeal mask airway (ILMA) are essential in the difficult airway algorithm for maintaining a patient’s oxygenation and can be applicable in patients with unanticipated difficult intubation [17]. As tracheal intubation is mandatory for operation, the ILMA has been reported as an effective technique with a high success rate [18,19].

Reports have evaluated the effectiveness of video laryngoscopes in obese patients requiring intubation. In previous studies regarding the efficacies of multiple laryngoscopes in obese patients, the results have been inconsistent. Although no studies have compared the McCoy laryngoscope and the C-MAC D-blade video laryngoscope, some studies have compared the GlideScope (a video laryngoscope) with the conventional Macintosh laryngoscope or McCoy laryngoscope; the intubation time was prolonged in the GlideScope group [20,21], but the IDS index was improved [20]. However, another study showed that the intubation time was lower with the GlideScope than with the Macintosh or McCoy laryngoscope [22]. Despite this, the conditions in those studies varied with respect to the characteristics of enrolled patients (e.g., BMI > 30 kg/m^2^ vs. BMI > 35 kg/m^2^) and the definition of intubation time including the starting point (time from grabbing the laryngoscope vs. time of laryngoscope blade passage between the teeth); they also varied with respect to the confirmation of intubation (until capnography was observed on the monitor vs. until the endotracheal tube was inserted into the trachea) [20,21,22]. Successful intubation was usually confirmed via capnography; however, a 1.6–5.1 s time delay with respect to the actual time may have occurred because of variations in capnometer systems and/or sampling line durations [23]. The conflicting results among studies of intubation in obese patients may also be the result of factors such as the BMI of enrolled patients, the definition of intubation time, and/or the skill of the practitioners.

To our knowledge, few studies have explored the effectiveness of the C-MAC video laryngoscope in obese patients. The anesthesiologist reported no subjective differences in intubation time or intubation difficulty between the C-MAC video laryngoscope and the Macintosh blade; however, further alternative techniques were needed in the Macintosh group [24]. One pilot study evaluated the C-MAC blade and D-blade of the C-MAC video laryngoscope in severely obese patients with a BMI > 40 kg/m^2^; they did not show any differences with respect to vocal cord exposure time, intubation time, or number of intubation attempts [25]. The findings of previous studies suggest that, compared with direct laryngoscopes, video laryngoscopes do not substantially reduce the total intubation time in obese patients; this conclusion is consistent with the results of the present study.

We examined possible predictors of difficult intubation in the current investigation. A normal mouth opening is measured by inserting three fingers into the oral cavity, which is approximately 4–6 cm. However, a limited mouth opening is linked to challenging laryngoscopy [14]. One predicted factor for challenging intubation is a thyromental distance of fewer than three finger lengths [26,27]. Despite the fact that the Mallampati score’s positive predictive value varied across multiple studies [26], it is a commonly used and easily applied measure, and a Mallampati score of III or IV has been linked to difficult intubations [28,29]. Though the limitations in prognathism have a low predictive value, they can be used in conjunction with other predictors [14,30]. A neck circumference of more than 40 cm has been linked to challenging intubation, particularly in patients who are obese [31]. Additionally, 24% of problematic intubations had a history of documented difficult intubation [32]. Not one of the predictors that we measured differed between the two groups.

According to the definition proposed by the American Association of Anesthesiologists, “difficult tracheal intubation” requires multiple attempts with or without any tracheal pathology [17]. A difficult laryngoscopy is commonly described by its Cormack–Lehane grade; however, this grade is not necessarily associated with difficult intubation [33]. Difficult intubation can also be described using the visual analog scale; however, this tends to be a more subjective clinical indicator with vague implications concerning intubation difficulty. Adnet et al. devised the IDS to describe “difficult intubation”, which was combined with objective indicators (e.g., number of intubation attempts, operators, or alternative techniques); the IDS index has been associated with intubation time [16], thus providing a useful assessment for the difficult intubation of obese patients [34]. In the present study, the IDS median values were 2 in the McCoy group and 1 in the C-MAC group, and the number of patients with an IDS of 0 was lower in the C-MAC group (McCoy, *n* = 8 vs. C-MAC, *n* = 16); these findings suggested that the C-MAC D-blade video laryngoscope facilitated the tracheal intubation of obese patients, compared with the McCoy laryngoscope. Among IDS indices, lifting force during laryngoscopy was needed more often in the McCoy group, whereas the POGO score and Cormack–Lehane grade did not show any differences between the two groups. More force may be needed for clinicians to maintain an adequate laryngoscopic view with the McCoy laryngoscope than with the C-MAC D-blade video laryngoscope. This premise is consistent with the results of a previous study, in which the IDS and proportion of increased lifting force were lower in the video laryngoscope group than in the direct laryngoscope group [20].

The intubation time did not significantly differ between the two groups; however, the vocal cords could be found easily with the C-MAC D-blade video laryngoscope, and the number of successful intubations within 30 s was higher in the C-MAC group (C-MAC, *n* = 22 vs. McCoy, *n* = 15). Oxygen saturation in obese patients rapidly decreases during anesthesia-induced apnea events because of reduced functional reserve capacity, dependent lung atelectasis, and increased work necessary for breathing [1]. A previous study revealed that the time to SpO_2_ of 90% was ≤ 3 min in obese patients with a BMI > 40 kg/m^2^ after 5 min of preoxygenation; this interval was significantly shorter than the interval in patients with a normal BMI [35]. In the present study, most patients were intubated within 60 s (McCoy, 98.03% and C-MAC, 100%). Moreover, the excluded patients with an intubation time > 90 s in the McCoy group were successfully intubated within 3 min by other anesthesiologists using the C-MAC video laryngoscope, and saturation in all participating patients was maintained at > 90% during the procedure. Desaturation in obese patients during apnea periods may be exacerbated by insufficient preoxygenation or the presence of underlying lung pathologies. Although the total intubation time did not significantly differ between groups, the C-MAC D-blade video laryngoscope appeared to be more useful for confirming vocal cord positioning in obese patients.

Previous studies comparing direct laryngoscopes with video laryngoscopes in patients with a BMI over 35 kg/m^2^ found that the success rates of intubation were not significantly different, although there were instances of failed intubation with direct laryngoscopes [20,21,24]. One study found that the success rate of the direct laryngoscope group was much lower compared to the GlideScope group [36]. Overall, the success rate itself appeared to be unaffected by the type of laryngoscope in individuals with a BMI of more than 35 kg/m^2^. There was no statistically significant difference observed between the two groups in the current investigation, which aligns with the findings from earlier studies.

Among the hemodynamic values, we found that the MAP significantly changed during the intubation procedure; in particular, the MAP immediately after intubation was lower in the C-MAC group. We did not record the depth of anesthetic at the exact time prior to intubation. However, anesthesia was induced with an equivalent regimen, and we used a BIS monitor on all patients. All patients were intubated at a BIS of 40–60 to maintain acceptable an depth of anesthesia. Although there is no definite proof that the video laryngoscope can alleviate hemodynamic changes after intubation compared with conventional laryngoscopes [37,38,39], obesity may have influenced the results in the present study, considering that the BMI of participants in previous studies was < 30 kg/m^2^. The catecholamine and hemodynamic responses after the intubation procedure are caused by the application of force during laryngoscopy, rather than by the intubation itself [40]; thus, hypertension, arrhythmias, and/or the elevation of intracranial/intraocular pressure can occur during the procedure [41,42]. As previously stated, the difference we observed between groups in MAP immediately after intubation may have resulted from the higher proportion of increased lifting force in the McCoy group. Because cardiovascular responses associated with the intubation procedure can be detrimental in patients with decreased cardiovascular functional capacity [43], the C-MAC D-blade video laryngoscope can help to attenuate hemodynamic responses during laryngoscopy.

There were several limitations in this study. First, the anesthesiologists could not be blinded to the laryngoscopes used. Second, subjective scales (e.g., Cormack–Lehane grade and POGO score) were used to evaluate the laryngoscope effectiveness. We also used objective scales in the IDS, including the number of intubation attempts, operator, and alternative techniques, as well as lifting force and laryngeal pressure. Third, we excluded patients who required > 90 s of intubation in all groups. It would have been preferable if complicated cases were included in this study; however, a safety mechanism was required to protect potentially extremely complicated participants, and therefore, we chose not to include those patients in the analysis. Therefore, further studies with larger numbers of patients are needed to evaluate the advantages or limitations of video laryngoscope use in obese patients. Finally, we did not include a control group in which intubation was conducted using the Macintosh laryngoscope. Previous studies have shown that a BMI > 30 kg/m^2^ and a neck circumference > 43 cm are associated with difficult intubation, as indicated by an IDS > 5, when a Macintosh laryngoscope is used [44,45]. Patients in the present study were expected to exhibit difficult intubation because their BMIs were >35 kg/m^2^ and their mean neck circumference exceeded 45 cm. Importantly, we conducted this study without a control group to ensure that the airway was safely secured, rather than risking multiple intubation attempts that could lead to airway injury in patients with expected difficult intubation.

## 5. Conclusions

In conclusion, both the McCoy laryngoscope and the C-MAC D-blade video laryngoscope could be useful for obese patients with a BMI > 35 kg/m^2^ because intubation using both laryngoscopes was slightly difficult, with an IDS of 1–2. The C-MAC video laryngoscope, which could facilitate early vocal cord exposure, may be particularly helpful in obese patients who are susceptible to rapid desaturation during apnea events. Additionally, the C-MAC D-blade video laryngoscope is more appropriate than the McCoy laryngoscope for patients who require hemodynamic stability during the intubation procedure.

## Figures and Tables

**Table 1:** Mask ventilation scale.

Grade	Description
I	Ventilated using a mask
II	Ventilated using a mask with oral airway
III	Difficult mask ventilation (inadequate, unstable, or requiring two practitioners)
IV	Mask ventilation could not be achieved

As described by Han et al. [15].

**Table 2:** Intubation difficulty scale.

Parameter	Scores	Rules for Calculating IDS Score
Number of attempts	N1	N1: Each additional attempt adds 1 pt.
Number of operators	N2	N2: Each additional operator adds 1 pt.
Number of alternative techniques	N3	N3: Each alternative technique adds 1 pt. (e.g., patient repositioning or change in materials used)
Cormack grade 1	N4	N4: Apply Cormack grade for the first oral attempt.
Lifting force required		
Normal/increased	N5 = 0/N5 = 1	
Laryngeal pressure		
Not applied/applied	N6 = 0/N6 = 1	
Vocal cord mobility		
Abduction/adduction	N7 = 0/N7 = 1	
Total IDS = Sum of scores	N1–N7	
IDS score	Degree of difficulty	
0	Easy	
0 < IDS ≤ 5	Slight	
5 < IDS	Moderate to major	

As described by Adnet et al. [16]. IDS, intubation difficulty scale.

**Table 3:** Patient demographic data and airway assessment characteristics.

Characteristics	Laryngoscope		*p*-Value
	McCoy Group (*n* = 51)	C-MAC Group (*n* = 51)	
Age (years)	43.76 ± 12.94	39.90 ± 10.87	0.103
Sex (M/F) (*n*)	19/32	26/25	0.163
Mouth opening (cm)	5 (4.5–5)	5 (5–5)	0.080
Mallampati classification (*n*)			
I/II/III/IV	3/26/20/2	6/26/16/3	0.649
Head/neck movement (*n*)			
>90°/90°/<90°	33/8/10	34/5/12	0.641
Ability to prognath (*n*)			
Yes/partial/no	42/9/0	44/6/1	0.439
Weight (kg)	102.24 ± 14.76	107.21 ± 15.64	0.101
Thyromental distance (cm)	8 (7–9)	9 (8–10)	0.083
Body mass index (kg/m^2^)	36.75 (35.5–39.75)	36.53 (35.6–38.87)	0.878
History of difficult intubation (*n*)			
None/questionable/definite	26/25/0	17/34/0	0.071
Neck circumference (cm)	45.59 ± 4.51	46.02 ± 4.43	0.627
ASA class (II/III) (*n*)	42/9	41/10	0.799

Data are shown as mean ± standard deviation, median (interquartile range), or numbers. ASA, American Society of Anesthesiologists.

**Table 4:** Comparison of intubation data between McCoy and C-MAC groups.

	Laryngoscope		
Outcomes	McCoy Group (*n* = 51)	C-MAC Group (*n* = 51)	*p*-Value
Mask ventilation			0.070
I/II/III/IV	33/15/3/0	27/13/11/0	
Vocal cord exposure time (s)	5.85 (4.81–8.10)	4.14 (3.47–5.52)	<0.001 *
Vocal cord passage time (s)	10.76 (8.75–14.39)	10.33 (9.21–14.35)	0.756
Intubation time (s)	32.54 (29.70–37.21)	30.72 (28.12–32.19)	0.499
POGO score (%)	70 (50–80)	70 (50–90)	0.529
IDS	2 (1–3)	1 (0–2)	0.046 *
Teeth or lip trauma (*n*)	0	2	0.153

Data are shown as median (interquartile range) or numbers. POGO, percentage of glottis opening. * *p* value < 0.05

**Table 5:** Comparison of IDS between McCoy and C-MAC groups.

	Laryngoscope		
Outcomes	McCoy Group (*n* = 51)	C-MAC Group (*n* = 51)	*p*-Value
Number of attempts (1/2)	49/2	51/0	0.495
Number of operators (1/2)	50/1	51/0	0.315
Number of alternative techniques	0	0	NA
Cormack grade			
I/II/III/IV	12/33/5/1	19/30/2/0	0.260
I,II/III,IV	45/6	49/2	0.269
Lifting force required			
Normal/increased	35/16	24/27	0.027 *
Laryngeal pressure			
Not applied/applied	15/36	11/40	0.363
Vocal cord mobility			
Abduction/adduction	51/0	51/0	NA

NA, not applicable. * *p* value < 0.05

**Table 6:** Hemodynamic variables.

Variables	Group	Anesthesia Time				*p*-Value
		Before Induction	Immediately before Intubation	Immediately after Intubation	3 Min after Intubation	
MAP (mmHg)	McCoy	110.96 ± 13.82	76.96 ± 11.24	105.20 ± 22.40	82.12 ± 14.23	0.042 *
	C-MAC	105.78 ± 19.43	76.02 ± 9.48	95.02 ± 16.79	78.31 ± 11.43	
HR (beats/min)	McCoy	79.92 ± 17.93	76.35 ± 15.81	88.90 ± 12.73	82.14 ± 12.66	0.974
	C-MAC	77.92 ± 13.89	75.82 ± 15.06	87.88 ± 14.62	81.43 ± 12.93	
SpO_2_ (%)	McCoy	97 (96–98)	100 (99–100)	99 (99–100)	98 (97–99)	0.775
	C-MAC	97 (96–98)	99 (99–100)	99 (98–100)	98 (98–99)	

Data are shown as mean ± standard deviation or median (interquartile range). MAP, mean arterial pressure; HR, heart rate. * *p* value < 0.05

## Data Availability

The data utilized and examined in the current investigation can be obtained from the corresponding author due to the inclusion of individualized information.
